# Impact of carbon inputs on soil carbon fractionation, sequestration and biological responses under major nutrient management practices for rice-wheat cropping systems

**DOI:** 10.1038/s41598-019-45534-z

**Published:** 2019-06-24

**Authors:** Ajay Kumar Bhardwaj, Deepika Rajwar, Uttam Kumar Mandal, Sharif Ahamad, Bhumija Kaphaliya, Paramjit Singh Minhas, Mathyam Prabhakar, Rakesh Banyal, Ranbir Singh, Suresh Kumar Chaudhari, Parbodh Chander Sharma

**Affiliations:** 1 ICAR-Central Soil Salinity Research Institute (CSSRI), Karnal, 132001 Haryana India; 2 CSSRI Regional Research Station, Canning Town, 743329 West Bengal India; 3 ICAR-Central Research Institute for Dryland Agriculture, Hyderabad, 500059 Telangana India; 4 Krishi Anusandhan Bhawan –II, Pusa, New Delhi 110012 India

**Keywords:** Environmental impact, Carbon cycle

## Abstract

Major nutrient management systems for rice-wheat cropping were compared for their potential to credit organic carbon (C) to the soil, its fractionation into active (very labile, VLc; labile, Lc) and passive (less labile, LLc; non-labile, NLc) pools, and crop yield responses. A ten-year long experiment was used to study effects of: (i) no inputs (Control, O), (ii) 100% inorganic fertilizers (F) compared to reduced fertilizers inputs (55%) supplemented with biomass incorporation from (iii) opportunity legume crop (*Vigna radiata*) (LE), (iv) green manure (*Sesbania aculeata*) (GM), (v) farmyard manure (FYM), (vi) wheat stubble (WS), and (vii) rice stubble (RS). Maximum C input to soil (as the percentage of C assimilated in the system) was in GM (36%) followed by RS (34%), WS (33%), LE (24%), and FYM (21%) compared to O (15%) and F (15%). Total C input to soil had a direct effect on soil C stock, soil C fractions (maximum in VLc and LLc), yet the responses in terms of biological yield were controlled by the quality of the biomass (C:N ratio, decomposition, *etc.*) incorporated. Legume-based biomass inputs accrued most benefits for soil C sequestration and biological productivity.

## Introduction

Soil carbon (C) is not only considered as the most logical sink for atmospheric CO_2_ but it is also an important soil quality component playing role in controlling soil fertility and crop production, hydrology and drainage, greenhouse gas emissions, and several other ecosystem functions on earth^1–5^. Whereas at global scale increasing soil C has been recognized as a major strategy for curtailing increased carbon dioxide (CO_2_) levels in earth’s atmosphere^6,7^, at the farm level increasing soil C is crucial for sustaining the quality and productivity of soil^8^. In the Indo-Gangetic plain, cultivated lands are under intensive pressure to meet the food demand of the exploding population of India. The plain is one of the most intensively cultivated areas in the world, feeding 50% of India’s population with only 13% share in the total geographical area^9^. Rice (*Oryza sativa*) in late summer/monsoon season followed by wheat (*Triticum aestivum*) in the winter season is the major cropping system with highly intensive soil and crop management operations^10,11^. The alluvial soils of the region have fairly high annual productivity in terms of the C assimilated from the atmosphere into crop biomass, both above and below the ground^12^.

While the C assimilated in the above-ground biomass largely leaves farm gate in the form of grain and straw harvest, the C added to soil as root biomass and rhizodeposition or as soil amendment stays longer, depending upon the soil management. The soil C contributes to nutrient enrichment of the soil, their availability to crop plants and soil quality improvement. Increase in soil C can be linked to many other improved ecosystem functions^8^. Nutrient management through fertilizers is a key operation in crop production. Yet the indiscriminate or sole use of inorganic fertilizers has led to the deterioration of soil health. Rice-wheat systems are highly intensive cropping systems, in terms of energy requirement and nutrient removal, in South Asia. Therefore, integrated nutrient management (INM) practices with the combined use of inorganic and organic fertilizers/manures have long been advocated to farmers with a goal to improve soil health and ecosystem services. With time, different nutrient and carbon management systems have evolved namely, green manuring with *Sesbania aculeata*, use of legumes as opportunity crops after wheat harvest and before rice transplanting, incorporation of farmyard manure, and practice of retaining and incorporating crop residues into the soil. Though the C inputs in these managements may be different along with differences in the quality of organic matter added, each one of them has evolved as a complete package of practices for crop nutrient management and sustainable crop production. In green manuring, a legume crop (*Sesbania aculeata*) is taken up during the lean period between wheat harvest and rice planting. Similarly, green gram (*Vigna radiata*) can be accommodated as an opportunity crop after wheat harvest and before rice transplanting and its biomass can be incorporated into the soil. More recently, cereal crop residue/stubble retention and its incorporation into soil have been advocated to the farmers to avoid burning of crop stubble and residues, so as to build up soil C and to curtail air pollution in the region. While the quantitative differences in organic matter inputs into the soil (incorporated crop residues, *in-situ* amendments, *ex-situ* additions, root biomass, and rhizodeposition) are anticipated to result in differences in soil C, the qualitative differences (C:N ratio, decomposability, etc.) might play role in C fractionation in soil. Carbon fractionation into active and passive fractions has an important role in C sequestration in soil^11–13^.

Several studies have evaluated the inherent benefits of such management practices in terms of nutrient supply and carbon sequestration. Yet, long-term side by side comparisons of these systems in terms of total C balance including, C assimilated, C retained/incorporated in the soil, and changes in active and passive fractions of soil C are lacking. Most importantly, biological responses of these unique C balances have often been overlooked under these nutrient management systems, particularly in terms of qualitative and quantitative differences in organic matter added. Majumdar *et al*.^13^ reported that inorganic fertilizers are able to just maintain the soil organic C content, while inorganic fertilizers plus organics increased soil organic C in rice-wheat systems. In rice-wheat system, soil organic C concentration in surface 0.6 m was higher (1.8–6.2 g kg^−1^) in FYM-treated soils compared with 1.7–5.3 g kg^−1^ in NPK, and 0.9–3.0 g kg^−1^ in unfertilized plots^14^. Ghosh *et al*.^15^ studied the effect of organic and inorganic amendments on soil carbon sequestration potential in long term fertility experiment (19 years) in the plains of eastern India and reported that cultivation without organic amendments depleted total C content by 39–43% when compared with the addition of organic amendments. Wei *et al*.^16^ also suggested that crop straw incorporation with chemical N, P, K fertilizers is a better soil fertilization practice in the light sandy loam soil of the Huang-Huai-Hai Plain of China compared to their sole use. Effects of C returned to the soil in the form of wheat straw, farmyard manure, green manure, rice straw, *etc*. on soil organic C, grain yield, physical properties, organic matter and total N content of the soil, total organic C and plant growth have been thoroughly investigated^17–22^. So far, the notion is that organic matter recycling or biomass incorporation into the soil in intensively managed agricultural systems is driven by the net inputs of organic matter only. Therefore, most studies on residue-based or organically managed systems fall short of relating C management to the quality of organic matter inputs and their biological responses, especially in intensive rice-wheat systems.

The aim of this study was to budget C assimilation, C input into soil, partitioning of this C into active and passive soil C fractions, determining organic C sequestration potential of these management practices, and relating changes in these parameters to organic matter quality, net inputs and also their biological responses, over a long-term period in rice-wheat cropping system.

## Results

### C assimilated and incorporated into the soil

**Figure 1:**
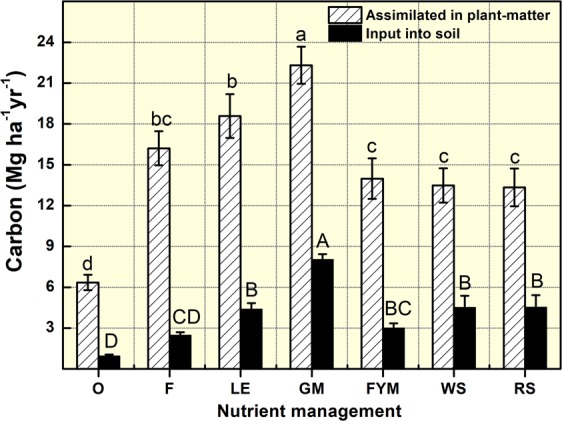
Carbon (C) assimilated in the nutrient management systems and C input (*ex situ* + *in situ*) into the soil. O = no fertilizer, F = 100% inorganic fertilizers, LE = opportunity legume crop (*Vigna radiata*), GM = green manuring, FYM = farmyard manure, WS = wheat stubble, RS = rice stubble. Error bars denote ± 1 SE. Treatments with same letters are not significantly different at p < 0.05.

Budgeting total plant assimilated C and C-input into soil, revealed that the maximum C was assimilated in GM (22.3 Mg ha^−1^) followed by LE (18.6 Mg ha^−1^), F (16.2 Mg ha^−1^), FYM (14.0 Mg ha^−1^), WS (13.5 Mg ha^−1^), RS (13.3 Mg ha^−1^), and O (6.3 Mg ha^−1^) (Fig. 1). Carbon input into the soil also varied in the same order. The maximum input of C into soil was in GM (8.0 Mg ha^−1^) followed by WS (4.5 Mg ha^−1^), RS (4.5 Mg ha^−1^), LE (4.4 Mg ha^−1^), FYM (3.0 Mg ha^−1^), F (2.5 Mg ha^−1^) and O (0.9 Mg ha^−1^). The C input into the soil as the percentage of C assimilated in the system was maximum in GM (36%) and least in O and F (15%).

### Soil carbon and its fractions

**Figure 2:**
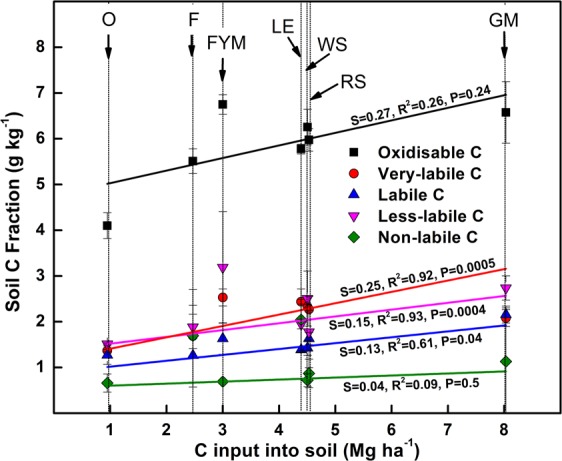
Relationship between carbon (C) input into the soil and soil C fractions under different nutrient management. O = no fertilizer, F = 100% inorganic fertilizers, LE = opportunity legume crop (*Vigna radiata*), GM = green manuring, FYM = farmyard manure, WS = wheat stubble, RS = rice stubble. Error bars denote ± 1 SE.

Oxidizable C was the maximum in FYM followed by GM and crop residue (WS, RS) treatments in the surface 0.15 m soil (Table 1). At the lower depths (0.15–0.30 m), there was no significant difference in the oxidizable C for any management. At both depths O and F accumulated least oxidizable C. VLc (very labile C) and LLc (less labile C) fractions constituted a major part of soil organic C, for all managements. All integrated nutrient management (INM) accumulated a similar amount of VLc fraction for all measured depths. GM accumulated the maximum Lc fraction at the surface 0–0.15 m. The LLc fraction was maximum in FYM which was followed by all other integrated nutrient managements. Management O had least LLc fraction in surface 0.15 m. There was no difference in NLc fraction for any of the treatments, for any measured depth. There was 46 to 65% decrease in oxidizable C, from the surface (0–0.15 m) to lower layer (0.15–0.30 m). Change in soil C content was directly related to the C input to the soil (Fig. 2). In general, the most increases were in the VLc and the LLc fractions of soil C in all management. With an increase in C input, the most significant increase was noticed in the Lc (labile C) and the LLc (less labile C) fractions. Management FYM had maximum contributions to the LLc and the VLc fractions while GM had a maximum contribution to Lc. Non-labile (NLc) C fraction changed little with increased total C input to the soil in different treatments.

**Table 1:** Depth-wise distribution of soil carbon fractions under different nutrient management.

Soil depth (m)	Management	TC	OC	Soil carbon Fractions			
				VLc	Lc	LLc	NLc
		(g kg^−1^)	(g kg^−1^)	(g kg^−1^)	(g kg^−1^)	(g kg^−1^)	(g kg^−1^)
0–0.15	O	4.81^D^	4.10^D^	1.37 ^C^	1.27^B^	1.51^B^	0.66 ^A^
	F	6.54 ^C^	5.51 ^C^	1.69^BC^	1.26^B^	1.89^AB^	1.70 ^A^
	LE	7.83^AB^	5.78^BC^	2.44 ^A^	1.39^B^	1.95^AB^	2.05 ^A^
	GM	8.11 ^A^	6.58^AB^	2.08^AB^	2.16 ^A^	2.74^AB^	1.13 ^A^
	FYM	8.04 ^A^	6.75 ^A^	2.53 ^A^	1.63^AB^	3.19 ^A^	0.69 ^A^
	WS	6.99^BC^	6.26^ABC^	2.33 ^A^	1.43^AB^	2.51^AB^	0.72 ^A^
	RS	6.55 ^C^	5.98^ABC^	2.27 ^A^	1.63^AB^	1.78^AB^	0.87 ^A^
0.15–0.30	O	3.27 ^C^	2.21 ^A^	1.28^B^	0.35 ^A^	0.59 ^A^	1.05 ^A^
	F	3.46^BC^	2.59 ^A^	1.76 ^A^	0.30 ^A^	0.52 ^A^	0.88 ^A^
	LE	3.59^ABC^	2.67 ^A^	1.52^AB^	0.47 ^A^	0.68 ^A^	0.92 ^A^
	GM	3.94 ^A^	2.59 ^A^	1.58^AB^	0.45 ^A^	0.56 ^A^	1.35 ^A^
	FYM	3.32 ^C^	2.35 ^A^	1.46^AB^	0.56 ^A^	0.49 ^A^	0.81 ^A^
	WS	3.83^AB^	2.57 ^A^	1.54^AB^	0.53 ^A^	0.49 ^A^	1.27 ^A^
	RS	3.68^ABC^	2.55 ^A^	1.69 ^A^	0.34 ^A^	0.52 ^A^	1.13 ^A^

OC, oxidizable carbon (C); TC, total C; VLc, very labile C fraction; Lc, labile C fraction; LLc, less labile C fraction; NLc, non-labile C fraction. O = no fertilizer, F = 100% inorganic fertilizers, LE = opportunity legume crop (*Vigna radiata*), GM = green manuring, FYM = farmyard manure, WS = wheat stubble, RS = rice stubble. For each soil depth, numbers in a column with the same alphabet are not significantly different (p < 0.05).

### Organic C sequestration potential and stock

**Figure 3:**
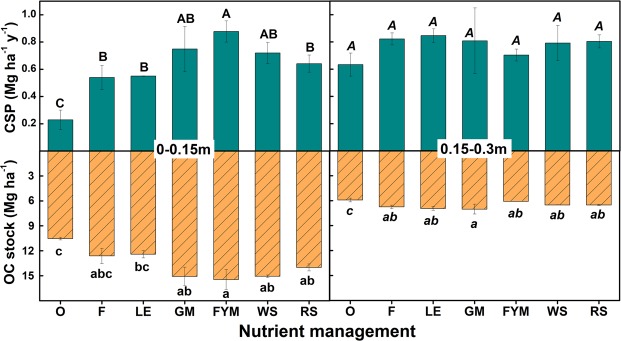
Carbon (C) sequestration potential (CSP) and soil C stock under different nutrient management after 10 years of initiation (2005–2015). O = no fertilizer, F = 100% inorganic fertilizers, LE = opportunity legume crop (*Vigna radiata*), GM = green manuring, FYM = farmyard manure, WS = wheat stubble, RS = rice stubble, OC = organic carbon, CSP = carbon sequestration potential. Error bars denote ± 1 SE. Nutrient management with same letters are not significantly different at p < 0.05.

Significantly higher carbon sequestration potential (CSP) was noted for FYM, GM and WS management, for shallower depths (0–0.15 m) (Fig. 3). For lower depths (0.15–0.30 m), there were no significant differences amongst management. Different managements and C inputs led to significant variation in bulk density only for surface 0.15 m soil (Fig. S1). LE and GM had the least bulk density at this depth while there were no significant differences at 0.15–0.30 m. Consequent of C inputs and bulk density changes, soil C stock was maximum in FYM, GM, and WS for 0.15 m. For all depths, O had the least organic C stock.

### Biological responses to C balance

**Figure 4:**
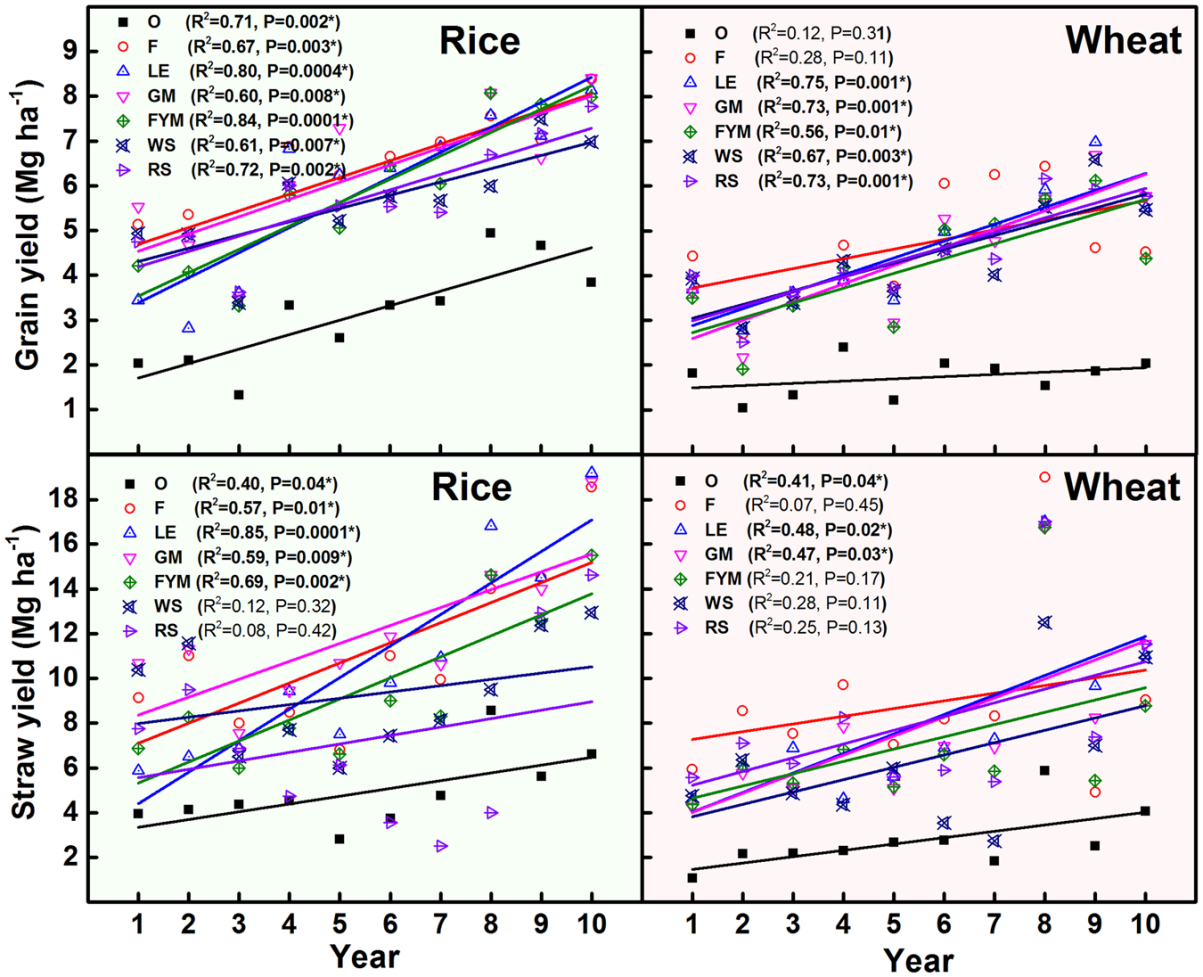
Trends in grain and straw yield of rice-wheat for 10 years (2005–2015) of nutrient management. O = no fertilizer, F = 100% inorganic fertilizers, LE = opportunity legume crop (*Vigna radiata*), GM = green manuring, FYM = farmyard manure, WS = wheat stubble, RS = rice stubble.

**Figure 5:**
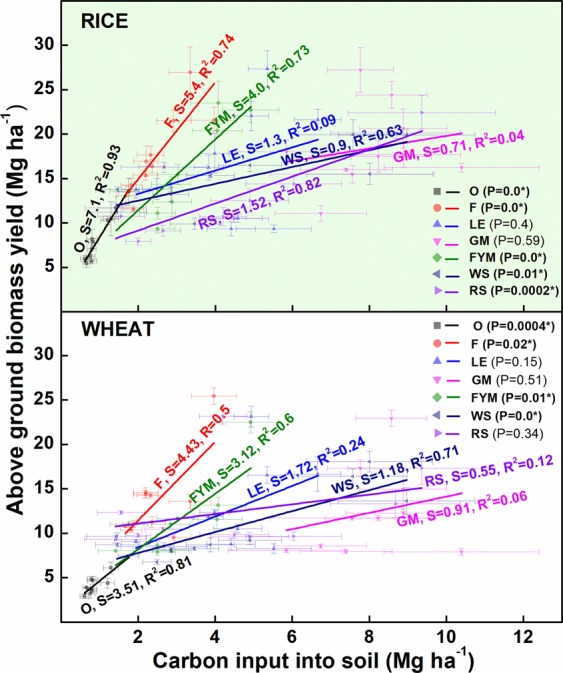
Response of carbon (C) input into the soil in terms of above ground biomass yield (grain + straw), under different nutrient management. O = no fertilizer, F = 100% inorganic fertilizers, LE = opportunity legume crop (*Vigna radiata*), GM = green manuring, FYM = farmyard manure, WS = wheat stubble, RS = rice stubble. Horizontal error bars denote ± 1 SD for C input into the soil and vertical error bars denote ± 1 SD for yield.

There were significant changes in grain and straw yield over a ten year period (Fig. 4). In case of rice, GM consistently produced the maximum yield (grain as well as straw) over the years along with F (100% inorganic fertilizers). Farmyard manure (FYM) application and LE trailed during the initial 5 years yet matched F and GM during 6–10 years. Crop residue based management (WS, RS) consistently trailed behind in terms of grain and straw yield compared to other management. In wheat crop, F exceeded all other managements for the initial 6 years in terms of both grain and straw yield. Green gram (*Vigna radiata*) biomass incorporation (LE) and green manuring (GM) matched or even exceeded F management after 5–6 years. Thus, there was a breakeven period of ~ 5 years for organic amended management compared to F. Significant correlations were observed between C input and above ground biomass yield in all management but the degree of response varied for rice and wheat crop (Fig. 5). In the case of rice, all management had a significantly positive correlation between annual C input into soil and yield, except GM and LE. In case of the wheat crop, besides LE and GM, RS also had non-significant relations with C inputs. In general, the response in terms of total biological yield (grain + straw) decreased as the total C input into the soil increased.

## Discussion

Six major integrated nutrient management (INM) systems practiced in Indo-Gangetic plain were budgeted for carbon (C) assimilation, C input into soil and soil C sequestration over a 10 year period. Overall, the results suggested that the differences in soil carbon fractionation (active and passive pools) were driven by net inputs of C as well as the quality of biomass added in each management. The less labile (LLc) and the very labile (VLc) fractions were the most contributed to and there was hardly any change in non-labile fractions with increased inputs of C. The key differences in C inputs into the soil under these nutrient management systems were due to the above ground additions of organic matter. The maximum C input (*in-situ and ex-situ*) was in GM (green manure) followed by LE (Legume crop biomass incorporation), WS (wheat stubble retention), RS (rice stubble retention), F (100% inorganic fertilizers), and O (no fertilizers), in the decreasing order.

In terms of net C input into the soil, GM contributed the highest organic C in the form of green manure (*Sesbania aculeata)* biomass incorporated into the soil. Second highest C inputs were in case of legume opportunity crop (*Vigna radiata*; LE), and crop residues (Rice stubble [RS], Wheat stubble [WS]). Though the net C inputs into the soil were similar in these three managements, LE biomass has narrower C:N ratio compared to crop residues (RS, WS) (Table S2). FYM closely followed the LE, RS and WS management in terms of net C input and only slightly exceeded F management. Thus based on net C input into the soil, there were four discrete groups (Group 1: GM; Group 2: LE, WS, RS; Group 3: FYM, F; Group 4: O). In terms of both quality and quantity of C inputs, there were six broad categories: GM with high C input and narrow carbon to nitrogen (C:N) ratio of organic matter; LE with lower C input and narrow C:N ratio of organic matter; crop residues based management (RS, WS) with broader C:N ratio of organic matter yet C inputs equal to LE (recalcitrant organic matter); FYM with even lower C input but highly decomposed organic matter; F with no *ex-situ* or *in-situ* C addition but only 100% inorganic fertilizers, and O without any fertilizer. Consequent upon these periodic additions of organic C for 10 years, significant changes in soil organic C and its fractions could be expected. Several earlier works have suggested a direct linkage between net C input and soil organic C in agro-ecosystems^23–25^.

Soil organic C stock was higher in the surface 0.15 m. The decrease was almost 50% in the lower 0.15–0.30 m soil depth despite the fact that the soil disturbance with tillage as deep as 20 cm, which was done in these managements using a power tiller can be expected to move organic matter deeper. Both rice and wheat crops have maximum root biomass concentrated in 0.15 m soil depth^26,27^. Thus, it could be concluded that most organic amendments and organic matter additions remained concentrated in 0.15 m depth despite tillage operations. Similar observations have been noted by Guo *et al*.^28^. It is also possible that because organic inputs were incorporated into the soil during puddling for rice transplanting, they being lighter fractions in a soil-water puddle would tend to concentrate at the surface. The tillage (dry plowing) for wheat sowing is not as intensive as puddling to mix the organic matter any further down.

Of the total soil organic carbon, active fractions (VLc and Lc) constituted almost 50% and are liable to be easily lost. But no significant differences were noted in the passive fractions (LLc and NLc) due to management, yet the LLc and the NLc were the most significant fractions in case of LE, GM, and FYM. Since these are the biomasses with low C:N ratio (Table S2) or decomposed as in case of FYM, it is a high possibility that the organic matter added to soil in LE, GM and FYM was much further down in the process of decomposition than in the crop residue-based management. Drinkwater *et al*.^29^ had similar observations wherein manure and legume-based systems had higher storage of soil carbon and nitrogen despite no quantitative differences in C inputs. The qualitative differences (C: N ratio, decomposability) contributed to differences in soil organic C stocks, thus Farmyard manure (FYM) based management involving the input of highly decomposed organic matter, with more stable C components, not only resulted in higher total C stock but also most of it was in less labile fraction (LLc). Legume-based managements (GM, LE) also had higher organic C stock at all depths. This disproves the conventional notion that biomass quality in agricultural systems may not play a significant role in C storage in soil.

While both high C input and high biomass N (as in GM) benefited crop productivity in rice crop, right from the early establishment of these systems, in case of wheat crop, break-even period for integrated nutrient management (LE, GM, FYM, WS, RS) and F (100% inorganic fertilizer) was much longer. Even for the rice crop, in case of LE and FYM, the breakeven period with F was 7–8 years. Rice is primarily a low land crop and therefore water is no constraint, ensuring an adequate nutrient release and availability to plant. But in case of the wheat crop where mostly water-limited conditions prevail, nutrient release and their availability to plants may be limited, especially in the integrated management^30^.

Farmers relate the benefits of any management practice to the responses in terms of yield. Many farmers in the region take land on lease, and their interest, therefore, is more in getting immediate economic gains rather than having a long-term interest in soil health. Unless C input into the soil is related to the gains in productivity, such practices would be hard to sell to farmers. Evaluation of such responses (C-input effects on crop yields) for intensive agricultural systems is rare. For rice crop, the biological yield correlated positively with C inputs into the soil except for GM and LE, wherein increased C input into soil did not reflect in terms of any benefit in yield. Both *Sesbania* and *Vigna* are nutrient rich (especially nitrogen) biomasses and under water-sufficient conditions, as in case of rice crop, favored sufficient release of nutrients (especially N) during decomposition. Averagely, *Sesbania* had 2.7% (w/w) and *Vigna* had 1.7% (w/w) nitrogen which provided around 141.8 and 87.9 Kg N ha^−1^, respectively, via incorporation of their biomass under GM and LE management, respectively. Therefore, perhaps there was no N limitation in these systems. Nitrogen limitation has been noted to be a major constraint for C decomposition process. Carbon decomposition has been found to increase with a decrease in the initial C:N ratio of the organic matter/substrate^31^. Nitrogen poor materials have been demonstrated to provide more-efficient N use and less-efficient C use than the N rich substrates.

In wheat crop too, all managements responded to C inputs into the soil in terms of increase in biological yield except GM and LE. But, the slope of fit, which is an indicator of change in yield per unit change (increase in this case) in C input into soil increased. Besides in wheat season, RS was also non-responsive. Under water-limited conditions (as in wheat), dissolution and movement of nutrients to plant roots is restricted. Rice stubble has better N concentration than wheat stubble, and therefore, perhaps, it also provided an advantage during wheat growth when water (as well as nutrient availability) is a constraint, compared to rice season. Therefore, the advantages obtained under water-sufficient conditions (as in rice) may not be applicable under water-deficient conditions (as in wheat). It also seemed that the responses of C input to soil were both chemical and physical otherwise FYM would have responded the same way as RS. It was also evident that the responses in terms of biomass yield per unit C input to soil decreased as the total C inputs to soil increased. O, F and FYM were the most responsive managements (under any condition) to C inputs into soil, indicating that such management would benefit most, at farmer’s level, from intervention leading to increased organic matter incorporation into the soil. These were the management with low C input to soil. Crop residue management and nutrient management programs should be targeted at this group of farmers. The second group was that of crop residues (RS, WS) wherein responses were very moderate. These systems had only a marginal increase in crop productivity with each increment of C input, perhaps due to N limitations since cereal crop residues are low in N and more recalcitrant type of organic material. The third class included the management wherein succulent type, low C: N ratio and N rich materials were added (LE, GM). These systems showed no response in water sufficient conditions (rice crop) and very little response under water-limited conditions (wheat crop), precisely pointing to the fact that the nitrogen availability could be a major limitation along with quantitative C inputs for any integrated nutrient management or crop residue management program to succeed.

## Conclusion

Our study revealed that the fractionation of incorporated carbon (C) under an integrated nutrient management practice for the rice-wheat system was driven by both quantitative as well as qualitative differences in the C inputs to the soil. LE (*Vigna* as opportunity crop between rice and wheat and its biomass incorporation) and GM (green manure crop *Sesbania* biomass incorporation) based management had high biomass incorporation into the soil, yet these materials also had high N and narrow C:N ratio. RS (rice stubble retention) and WS (wheat stubble retention) systems had lower C inputs as well as wider C:N ratio. Legume-based systems indicated higher benefits in terms of C sequestration. FYM constituted decomposed organic matter compared to others. In terms of above ground biomass yield of rice, GM outperformed other managements right from the start, and LE and FYM had a break-even period of around 4–5 years compared to 100% inorganic fertilizer based management (F). For wheat crop, all managements compared to F had a break-even period of 6–7 years. Nitrogen availability indeed played a significant role. Both *Sesbania* and *Vigna* are high nitrogen (low C:N ratio) crops. Increase in biological yield with increased C input/incorporation worked for high C:N ratio biomass incorporation, but yield under low C:N ratio biomass incorporation based system (GM, LE) remained unresponsive to changes in C input to soil C. Hence, not only quantitative C inputs, but also nutrient (particularly nitrogen) contributions through organic matter played important role. Legume-based management systems (GM, LE) are, therefore, the best management options for rice-wheat systems in Indo-Gangetic plain of India to enhance crops productivity and soil C sequestration.

## Materials and Methods

### Study site and experimental layout

A field experiment on integrated nutrient management in rice-wheat systems was initiated in 2005 on a soil with sandy loam texture at ICAR-Central Soil Salinity Research Institute (CSSRI), Karnal, India, located at 29.43°N and 76.58°E. The treatments were laid in 5 m × 4 m plots with four replications in completely randomized blocks. At the initiation of experiment the soils recorded a pH of 8.5, soil organic carbon equal to 2.1 g kg^−1^, and bulk density equal to 1.43 Mg m^−3^ (ref.^32^). The experimental area lies in a semi-arid sub-tropical climate zone with very hot summers and cool winters. The mean annual precipitation is 750 mm.

With the treatments imposed for 10 years, the soil carbon fractions were determined in 2015, after harvesting of wheat crop. The study included seven nutrient management treatments in total: five treatments with reduced (55%) inorganic fertilizer doses in combination with organic sources namely, LE- legume (*Vigna radiata*) as opportunity crop, GM-green manuring (*Sesbania aculeata*), FYM- farmyard manure incorporation, WS- wheat stubble retention and RS-rice stubble retention, compared to ‘100% inorganic fertilizers only’ (F) and ‘no fertilizer at all’ (O) treatment. The treatment details are provided in Table S1. The treatments were replicated in four completely randomized blocks. The annual cropping system which was followed consisted of rice (*Oryza sativa* L.) in the summer followed by wheat (*Triticum aestivum* L.) in the winter season. In all treatments, during rice season, the soil was dry tilled to incorporate the organic amendments into the soil at the start of the first week of July, and then puddled-in towards the end of the first week of July, every year. Both operations were done using power tiller. One-month-old seedlings of var. Pusa 44 were transplanted at recommended row spacing (20 cm). In wheat season, soils were first dry-tilled using power tiller and then hand-pulled plough was used to sow the seeds of var. HD2967 at recommended row spacing (15 cm). Wherever applicable nitrogen fertilizer applications were in 3 equal splits at t = 0, 21 and 42 days after transplanting (rice)/sowing (wheat). For nitrogen, phosphorus, and potassium fertilization urea, diammonium phosphate (DAP) and muriate of potash (MOP), respectively, were used at the time of transplanting/sowing. The recommended rates of inorganic fertilizers were 180, 26, and 42 Kg ha^−1^ for N, P and K, respectively, for both crops. Besides only in F treatment, ZnSO_4_ was also applied as micronutrient fertilizer at the recommended rate of 7 Kg ha^−1^ at the time of transplanting/sowing. Rice was transplanted in July and harvested in the last week of October, and wheat was sown in the second week of November and harvested in the last week of March, every year. In rice, fields were flooded for pudding and thereafter ~10 cm of standing water on the surface was maintained for first one month. For the second month, ~5 cm of surface flooding was maintained with irrigation every penultimate day, and for the third month, irrigations were given on aweekly basis and the soils were only saturated, with no standing water on the soil surface. Two weeks before harvesting irrigations were stopped. For wheat crop, 3–4 surface irrigations were given at around 1 month interval. These are standard irrigation management practices for rice-wheat systems in the region.

Following management schedules were followed for different treatments:

1. **O**: Rice followed by wheat was grown without any inorganic fertilizers or organic inputs.

2. **F**: Rice followed by wheat was grown with full recommended inorganic fertilizer input (N:P:K:Zn = 180:26:42:7 Kg ha^−1^). No organic inputs were provided.

3. **LE**: Legume (LE), *Vigna radiata*, was grown in the summer lean period between wheat and rice as an “opportunity crop”. The *Vigna* seeds were sown in the first week of May, after wheat harvest. In the first week of July (after ~ 60 days of sowing) pods were harvested and the remaining plant biomass was incorporated into the soil, before rice transplanting.

4. **GM**: A green manure crop, *Sesbania aculeata*, was grown in the lean period between wheat and rice. The green manure crop was sown on or around 20^th^ of May every year after wheat harvest. After 35–40 days of sowing, the green manure crop was incorporated into the soil, before rice transplanting.

5. **FYM**: Farmyard manure (FYM) at the rate of 10 Mg ha^−1^ was incorporated in the soil just before transplanting of rice.

6. **WS**: 30 cm standing stubble was retained at the time of harvesting of wheat. It was dry-plowed into the soil before rice transplanting in the first week of July.

7. **RS**: 30 cm standing stubble was retained at the time of harvesting of rice. It was dry-plowed into soil at the time of wheat sowing in 2^nd^ week of November.

### Soil and plant sampling

Soil samples for carbon determinations were drawn after wheat harvest. Samples were air-dried and sieved (0.5 mm) for further analysis. Plant samples were taken at the harvest of rice and wheat crops for C, N determinations and other chemical analyses. Sampling was done for all organic amendments for determination of total C incorporated at the time of amendment application.

### Soil organic carbon and its fractions

Oxidizable organic C in soil was determined by modified Walkley and Black’s rapid titration method^33,34^. In this method, 2 grams of sieved (0.5 mm) soil sample was taken in 250 ml conical flask. 10 ml of 1 N K_2_Cr_2_O_7_ and 20 ml of concentrated H_2_SO_4_ was added in the conical flasks. 100 ml of distilled water was added followed by 10 drops of diphenylamine indicator and 0.5 g of NaF. Excess of 1 N K_2_Cr_2_O_7_ was back titrated against N/2 ferrous ammonium sulfate. A blank was simultaneously run and soil organic C was calculated. Total carbon (TC) content of the soil samples was determined by dry combustion method using CHNS Elemental analyzer (Shimadzu Pvt. Ltd, Kyoto, Japan). Different fractions of soil organic C comprising very labile, labile, less labile and non-labile (recalcitrant) were determined by oxidation with 12 N, 18 N and 24 N H_2_SO_4_ (acid/aqueous ratios of 0.5:1, 1:1 and 2:1, respectively). Very labile (VLc) fraction was determined by reaction with 12 N H_2_SO_4_; labile carbon (Lc) was determined by calculating the difference in oxidizable C determined with 18 N and 12 N H_2_SO_4_ (18 N H_2_SO_4_ minus 12 N H_2_SO_4_); the less labile (LLc) fraction was determined by calculating the difference in oxidizable organic C extracted with 24 N and 18 N H_2_SO_4_. Non-labile (NLc) fraction was calculated as the difference between total oxidizable carbon and oxidizable carbon determined with 24 N H_2_SO_4_. VLc and Lc make an active pool of carbon while LLc and NLc make a passive pool of carbon^32^.

### Soil organic carbon stock

Total soil organic C stock was calculated using the following relation:

1 $${\rm{Soil}}\,{\rm{organic}}\,{\rm{C}}\,{\rm{stock}}\,{\rm{in}}\,{\rm{soil}}={\rm{soil}}\,{\rm{C}}\,{\rm{content}}\times {\rm{bulk}}\,{\rm{density}}\times {\rm{soil}}\,{\rm{depth}}$$

where, the soil C content is given in g C kg^−1^ soil, bulk density in Mg m^−3^, depth in m and soil organic C stock in kg m^−2^.

Metallic cores of known volume (v) were used to measure the soil bulk density. Soil samples were taken at the depth of 0–0.15 m, and 0.15–0.30 m, after the wheat harvest. Samples were oven dried at 105 °C for 24 h to obtain the dry weight of soil (w). The ratio of w and v in g/cm^3^ was used to determine bulk density^35^.

### Carbon sequestration potential

Carbon sequestration potential (CSP) was determined for different soil depths (0–0.15 m, 0.15–0.30 m) by measuring the soil organic C for respective depth using following equation^36^:

2 $${CSP}\,(t\,h{a}^{-1}y{r}^{-1})=\frac{({Soil}\,{organic}\,{C}\,{at}\,{n}\,{years}\,{of}\,{treatment}-{Soil}\,{organic}\,{C}\,{at}\,{n}={0})}{n}$$

where n = number of years.

### Above ground biomass yield

Above ground biomass yield was measured in terms of total above ground crop biomass at the end of each season. The total above ground biomass was separated into grain and straw yield for each treatment and replications.

### Plant assimilated C and C input to soil

Plant assimilated C was calculated for each crop for each season from 2005–2015 based on the above and below ground biomass accumulated and its equivalent in C. Root biomass for rice and wheat plants was measured only in 2013–2014 (9^th^ cropping cycle) and 2014–2015 (10^th^ cropping cycle), and its empirical relation with above ground biomass/straw was used to calculate coarse root biomass for rest of the years. Rhizodeposition was calculated using the estimates given by Jones *et al*.^37^ according to which 27% of C allocated to roots roughly accounts for rhizodeposition in cereals and grasses. The total C content of grain, straw and root biomass was determined with CHNS elemental analyzer (Shimadzu Pvt. Ltd., Kyoto, Japan) for the conversion of total mass to C content. C incorporated into the soil was calculated annually by measuring the entire C added to the soil in a treatment during full cropping cycle in the form of root biomass (including rhizodeposition), crop residue retained (and incorporated), *Vigna* (opportunity crop) biomass incorporated, *Sesbania* (green manure) biomass incorporated, and *ex-situ* additions. *Ex-situ* addition included C added to the soil through farmyard manure in FYM treatment.

### Statistical analysis

Statistical analysis of variance (ANOVA) was used to test all parameters. Separation of means was done by Tukey’s honestly significant difference test^38^. The Statistical analysis was done using JMP (2009; SAS Inc., Cary, NC, USA). The weighted least-square method was used in Origin v8.5 (OriginLab, Northampton, MA, USA) to apply linear regression between the measured parameters. The graphical representations were drawn using Origin v8.5 software. All tests were performed at a significance level of 0.05.

## Supplementary information

Supplementary Information

## Data Availability

The data supporting the findings in the manuscript is freely available from the corresponding author on reasonable request.
