# Supplementary material for: Impact of carbon inputs on soil carbon fractionation, sequestration and biological responses under major nutrient management practices for rice-wheat cropping systems
Source: Sci Rep. 2019 Jun 24;9:9114. doi: 10.1038/s41598-019-45534-z (PMC6591388; doi:10.1038/s41598-019-45534-z)
Supplement: Supplementary file 1 — Supplementary Information [file 41598_2019_45534_MOESM1_ESM.pdf]

Supplementary Information

Impact of carbon inputs on soil carbon fractionation, sequestration and biological responses under major nutrient management practices for rice-wheat cropping systems

Ajay Kumar Bhardwaj[*1], Deepika Rajwar[1], Uttam Kumar Mandal[2], Sharif Ahamad[1], Bhumija Kaphaliya[1], Paramjit Singh Minhas[1], Mathyam Prabhakar[3], Rakesh Banyal[1], Ranbir Singh[1], Suresh Kumar Chaudhari[4], Parbodh Chander Sharma[1]

[1] ICAR-Central Soil Salinity Research Institute (CSSRI), Karnal 132001, Haryana, India.
[2] CSSRI Regional Research Station, Canning Town 743329, West Bengal, India.
[3]ICAR-Central Research Institute for Dryland Agriculture, Hyderabad 500059, Telangana, India.
[4] Krishi Anusandhan Bhawan –II, Pusa, New Delhi 110012, India.

Corresponding author:
Ajay Kumar Bhardwaj, Principal Scientist
Address:
ICAR-Central Soil Salinity Research Institute, Kachhwa Road, Karnal 132001, Haryana, India.
Tel: (+91) 184-2209359, Fax: (+91) 184-2290480, E-mail: ak.bhardwaj@icar.gov.in, ajaykbhardwaj@gmail.com

Table S1. Fertilizer nutrients and organic inputs under different nutrient management systems.

**Nutrient management**	**Crop cycle**			**Fertilizer/ organic inputs**			
				**Rice (**at transplanting**)**		**Wheat (**at sowing**)**	
	**May to June**	**July to Oct**	**Nov to Apr**	**Inorganic Fertilizer (N: P: K) kg ha^{-1}**	**Organic**	**Inorganic Fertilizer (N: P: K: Zn) kg ha^{-1}**	**Organic**
**O**	Fallow	Rice	Wheat	-	-	-	-
**F**	Fallow	Rice	Wheat	180: 26: 42	-	180: 26: 42: 7	-
**LE**	Opportunity legume crop *(Vigna radiata)*	Rice	Wheat	100: 16: 28	Legume crop biomass	100: 16: 28: 0	-
**GM**	Green manure crop *(Sesbania aculeata)*	Rice	Wheat	100: 16: 28	Green manure biomass	100: 16: 28: 0	-
**FYM**	Fallow	Rice	Wheat	100: 16: 28	Farmyard manure (FYM)	100: 16: 28: 0	-
**WS**	Fallow	Rice	Wheat	100: 16: 28	Wheat stubble	100: 16: 28: 0	-
**RS**	Fallow	Rice	Wheat	100: 16: 28	-	100: 16: 28: 0	Rice stubble

Table S2. Carbon and nitrogen concentrations of different materials used as organic amendments under different nutrient management.

**Management**	**Organic amendment**	**Nitrogen concentration (%)**	**Carbon Concentration (%)**	**C:N**
**LE**	Green gram (*Vigna radiata*) biomass	1.71±0.02	47.78±1.07	27.95±0.77
**GM**	Green manure (*Sesbania esculeata*) biomass	2.65±0.02	42.32±1.13	15.99±0.32
**FYM**	Farmyard manure	0.56±0.04	31.36±0.92	56.53±4.36
**WS**	Wheat straw	0.27±0.01	41.90±0.55	157.78±7.77
**RS**	Rice straw	0.46±0.01	42.42±0.33	93.10±3.48

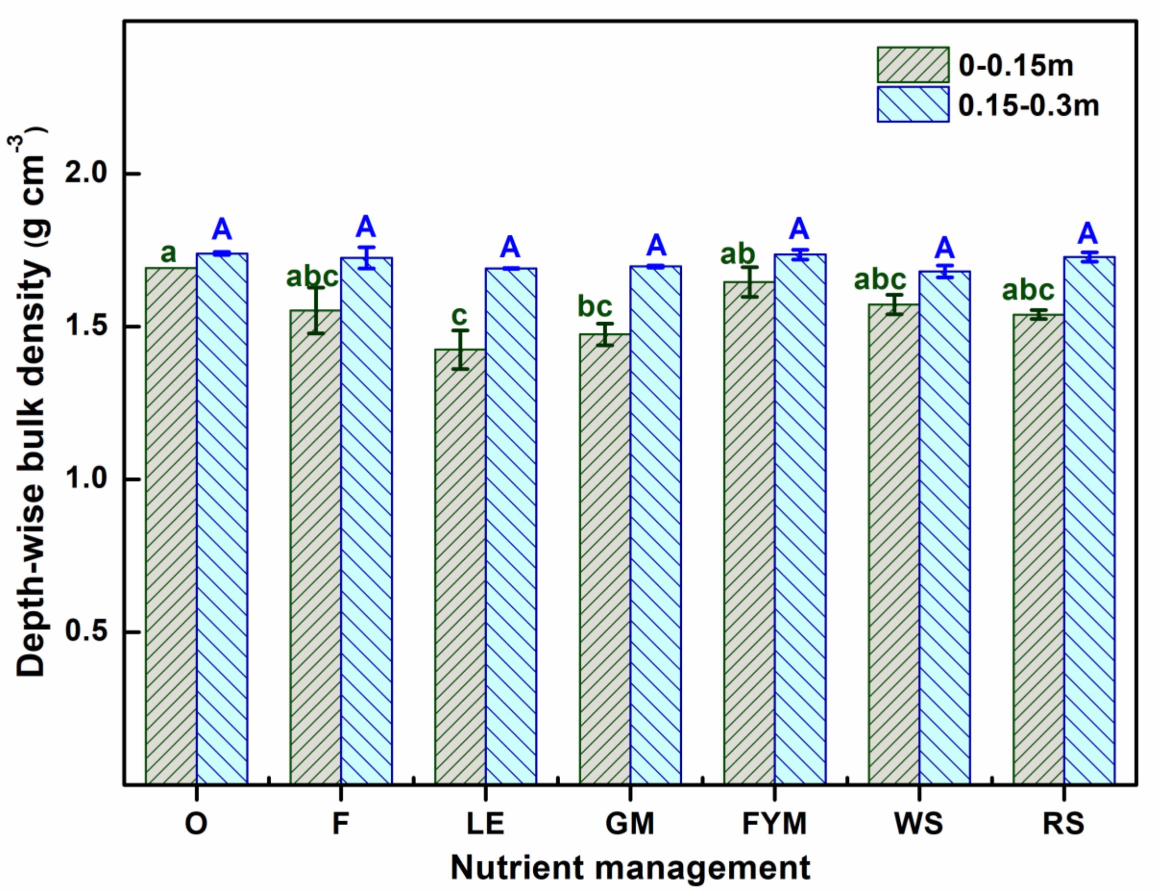

Fig. S1: Soil bulk density at different depths under different nutrient management after 10 years of initiation. O= no fertilizer, F=100% inorganic fertilizers, LE= opportunity legume crop (*Vigna radiata*) biomass incorporation, GM=green manuring, FYM=farmyard manure, WS= 30 cm wheat stubble retention and soil incorporation, RS= rice stubble retention and soil incorporation. Error bars denote ± 1 SE. Treatments with same letters are not significantly different at $p<0.05$.
